# In the absence of obstructive coronary artery disease, patients with class II and III obesity have decreased myocardial perfusion reserve

**DOI:** 10.1186/1532-429X-16-S1-P256

**Published:** 2014-01-16

**Authors:** W Patricia Bandettini, Li-Yueh Hsu, Hannah Conn, Susanne Winkler, Anders M Greve, Peter Kellman, O Julian Booker, Sujethra Vasu, Sujata M Shanbhag, Marcus Y Chen, Andrew E Arai

**Affiliations:** 1 Advanced Cardiovascular Imaging Laboratory, National Heart, Lung, and Blood Institute, National Institutes of Health, Bethesda, Maryland, USA; 2 Section of Cardiology, Wake Forest University School of Medicine, Winston-Salem, North Carolina, USA; 3 Department of Cardiology, University of Alabama, Birmingham, Alabama, USA

## Background

Obesity is associated with cardiovascular morbidity and mortality. This quantitative CMR perfusion study aims to examine the contribution of body mass index (BMI) to decreased myocardial perfusion reserve (MPR).

## Methods

123 patients with no obstructive epicardial coronary artery disease, defined by coronary computed tomographic angiogram demonstrating < 30% stenosis, underwent regadenoson CMR 1st-pass perfusion imaging, using 0.05 mmol/kg gadolinium (Gd) followed by rest perfusion imaging (also 0.05 mmol/kg Gd) performed 20 minutes later. The subjects were categorized into 4 groups: normal BMI (between 18.5-24.9 kg/m2, overweight (BMI 25-29.9 kg/m2), Class I obese (BMI 30-34.9), and Class II and III obese (BMI≥35). Myocardial blood flow (MBF) in ml/min/g and myocardial perfusion reserve (MPR) were quantified using a fully quantitative model constrained deconvolution.

## Results

**Figure 1:**
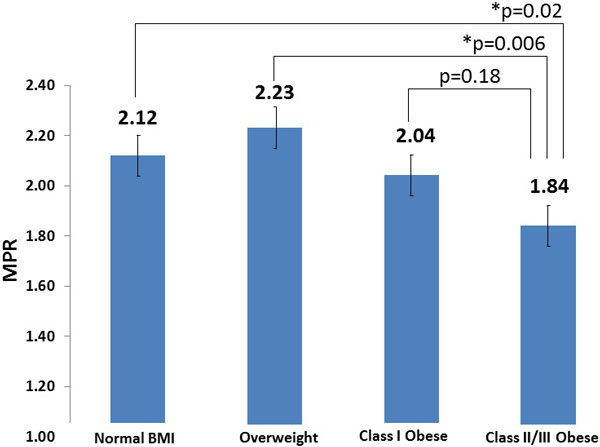
**Class II/III Obese subjects have a statistically significantly lower MPR compared to Normal BMI and Overweight groups**.

The normal BMI group had 25 patients (mean BMI 22.8 ± 0.3), the overweight group had 52 patients (mean BMI 27.4 ± 0.2), the Class I obese group had 22 patients (mean BMI 32.1 ± 0.3), and the Class II/III obese group had 24 patients (mean BMI 42.7 ± 1.1). MPR in the four groups was: Normal 2.12 ± 0.09, Overweight 2.23 ± 0.09, Class I Obese 2.04 ± 0.13, and Class II/III Obese 1.84 ± 0.11 (Figure 1). MPR was statistically higher in the Normal and Overweight groups compared to the Class II/III Obese group (p = 0.02 and 0.006 respectively).

## Conclusions

Even in the absence of obstructive coronary artery disease, Class II and III obesity is associated with decreased MPR as demonstrated by CMR quantitative measurement of regadenoson-rest perfusion.

## Funding

This research was supported by the Intramural Research Program of the National Heart, Lung, and Blood Institute, National Institutes of Health.

